# Long-term HbA1c trajectories and diabetes-related complications among adults with incident type 2 diabetes: A nationwide cohort study in Thailand

**DOI:** 10.1016/j.pmedr.2026.103693

**Published:** 2026-11-05

**Authors:** Anuchit Sermhattakit, Sawaeng Watcharathanakij

**Affiliations:** a Doctor of Philosophy Program in Pharmaceutical Sciences, Faculty of Pharmaceutical Sciences, Ubon Ratchathani University, Ubon Ratchathani, Thailand; b The Center of Pharmacoepidemiology and Data Analytics (PEpiDA), Division of Pharmacy Practice, Faculty of Pharmaceutical Sciences, Ubon Ratchathani University, Thailand

**Keywords:** Type 2 diabetes mellitus, HbA1c, Glycemic trajectories, Group-based trajectory modeling, Diabetes complications, Population health

## Abstract

**Objective:**

To identify 5-year HbA1c trajectories among adults with incident type 2 diabetes mellitus, factors associated with unfavorable trajectories, and associations with diabetes-related complications.

**Methods:**

We conducted a nationwide retrospective cohort study of 142,687 treated adults newly diagnosed with T2D in Thailand during 2017–2019, with follow-up through 2025. Group-based trajectory modeling identified HbA1c patterns. Multivariable multinomial and binary logistic regression assessed trajectory membership and microvascular and macrovascular complications, respectively. Missing covariates were addressed using multiple imputation. A three-year landmark analysis using Cox regression assessed associations with subsequent complications.

**Results:**

Four trajectories were identified: **Group 1, stable glycemic control (59.39%); Group 2, poor control with partial improvement (28.36%); Group 3, very poor control with deterioration (6.62%); and Group 4, persistently extremely poor control (5.63%).** Younger age, female sex, higher LDL-C, higher estimated glomerular filtration rate, and baseline insulin use were associated with unfavorable trajectories. Compared with Group 1, macrovascular complication odds increased across Groups 2–4 (adjusted odds ratios 1.23, 1.29, and 1.56, respectively). Microvascular complications were associated with Groups 2 and 4.

**Conclusions:**

Unfavorable HbA1c trajectories were common and associated with diabetes-related complications, particularly macrovascular disease. Longitudinal glycemic patterns may help identify populations requiring earlier preventive attention.

## Highlights

• Four five-year glycemic trajectories were identified among 142,687 adults.
• Over 40% of adults followed unfavorable glycemic trajectories.
• Younger age and baseline insulin use were linked to unfavorable trajectories.
• Macrovascular complication odds increased across unfavorable trajectory groups.
• Longitudinal glycemic patterns may inform population-level prevention.

## 1 Introduction

Type 2 diabetes mellitus (T2D) is a major and growing public health challenge worldwide and in Thailand, contributing substantially to microvascular and macrovascular complications, premature mortality, and healthcare burden (International Diabetes Federation, 2025; Puangpet et al., 2022; Farmaki et al., 2020). Glycated hemoglobin (HbA1c) is the standard measure for assessing glycemic control and guiding diabetes management (American Diabetes Association, 2024). However, conventional assessments based on single or average HbA1c values may not fully capture the dynamic nature of glycemic control over time. Individuals with similar HbA1c levels at diagnosis may subsequently follow markedly different patterns, including sustained control, improvement, deterioration, or persistent hyperglycemia (Luo et al., 2018; Venkataraman et al., 2017).

Previous studies in Thailand have primarily examined glycemic control using cross-sectional risk factors or clinical characteristics measured at a single time point (Puangpet et al., 2022; Techasuwanna et al., 2020; Preechasuk et al., 2022). Such approaches may overlook heterogeneity in long-term glycemic progression. Group-based trajectory modeling (GBTM) provides a person-centered approach for identifying subgroups of individuals who follow similar longitudinal patterns (Nguena Nguefack et al., 2020; Nagin, 2014). Studies using this approach have demonstrated distinct HbA1c trajectories among people with T2D, suggesting that longitudinal patterns may provide clinically relevant information beyond isolated HbA1c measurements (Luo et al., 2018; Venkataraman et al., 2017; Walraven et al., 2015). Nevertheless, evidence from large population-based cohorts in Asian settings remains limited, particularly regarding whether different long-term glycemic trajectories are associated with subsequent diabetes-related complications.

Understanding these longitudinal patterns at the population level may help identify groups with sustained glycemic vulnerability and inform earlier preventive strategies. Using nationwide routinely collected health data from Thailand, this study aimed to (1) identify distinct 5-year HbA1c trajectories among adults with incident T2D, (2) determine baseline demographic, clinical, and treatment factors associated with unfavorable trajectory membership, and (3) examine associations between trajectory groups and incident microvascular and macrovascular complications.

## 2 Methods

### 2.1 Study design and data source

This nationwide retrospective cohort study used longitudinal data from the Thai Ministry of Public Health's Health Data Center (HDC), which integrates patient records from public healthcare facilities nationwide through the standardized 43-file health data structure.

Outpatient and inpatient records were linked using de-identified IDs and newly encrypted IDs, with data-quality reviews conducted at hospital and provincial levels (Rittiphairoj et al., 2022; Peerawaranun et al., 2024). The database included 65,887,954 unique individuals during 2016–2025, reflecting broad population and geographical coverage. The study was reported in accordance with the Strengthening the Reporting of Observational Studies in Epidemiology (STROBE) statement (von Elm et al., 2007).

### 2.2 Study population

Adults aged ≥18 years newly diagnosed with T2D between January 1, 2017, and December 31, 2019, were identified using ICD-10 codes E11 and E11.9. With records available from January 2016, incident T2D was defined by a 12-month washout period without a recorded T2D diagnosis or glucose-lowering medication use. The index date was the first recorded T2D diagnosis. Individuals with type 1, gestational, or other specified diabetes were excluded.

Trajectory analysis required ≥5 years of follow-up, ≥ 4 valid HbA1c measurements, and at least one antidiabetic medication record at the index date. The ≥5-year follow-up enabled characterization of long-term HbA1c trajectories, while ≥4 measurements supported reliable trajectory estimation, consistent with previous longitudinal studies (Martinez-De la Torre et al., 2022; Li et al., 2023). The medication criterion improved the specificity of T2D identification and defined a pharmacologically treated incident cohort. Follow-up data extended through 2025.

### 2.3 HbA1c measurements and trajectory modeling

HbA1c measurements recorded during the first 5 years after the index date were used to identify longitudinal glycemic trajectories. When multiple HbA1c measurements were recorded on the same day, the mean value was used. Values <4.0% (20 mmol/mol) or > 18.0% (173 mmol/mol) were excluded as biologically implausible.

### 2.4 Baseline covariates

Baseline covariates included age, sex, body mass index (BMI), systolic and diastolic blood pressure (SBP and DBP), smoking status, alcohol use, estimated glomerular filtration rate (eGFR), low-density and high-density lipoprotein cholesterol (LDL-C and HDL—C), triglycerides, healthcare coverage, number of antidiabetic drug groups, and baseline insulin use. Baseline clinical and laboratory covariates were defined using measurements recorded within ±6 months of the index date. When multiple measurements were available within this window, the value closest to the index date was selected.

Comorbidity burden was categorized as 0, 1, or ≥ 2 documented conditions, including hypertension, dyslipidemia, chronic kidney disease, atherosclerotic cardiovascular disease, heart failure, cerebrovascular disease, obesity, and liver disease (Glasheen et al., 2019; Quan et al., 2011).

### 2.5 Diabetes-related complications

The study outcomes were incident microvascular and macrovascular complications, identified using International Classification of Diseases, Tenth Revision (ICD-10) diagnostic codes. Microvascular complications included diabetic nephropathy/chronic kidney disease (E11.2, N18.x), retinopathy (E11.3), neuropathy (E11.4), diabetic foot/peripheral angiopathy (E11.5), and other diabetes-related complications (E11.6–E11.7). Macrovascular complications included coronary artery disease/myocardial infarction (I20–I25), cerebrovascular disease (I60–I69), heart failure (I50), and peripheral arterial disease (I70.2, I73.9).

Complications were identified from routinely recorded ICD-10 diagnoses rather than systematic clinical screening, reflecting clinically recognized and documented events. To distinguish incident from pre-existing complications, diagnostic records from January 2016 through 1 year after the index date were reviewed. Complications recorded during this period were considered pre-existing and excluded from the corresponding outcome risk set. Incident complications were defined as new diagnoses occurring >1 year after the index date without a prior record of the corresponding complication (Harding et al., 2019; Bonora et al., 2020). The pre-index look-back period varied by index date because records were available only from January 2016 onward.

### 2.6 Statistical analysis

Group-based trajectory modeling (GBTM) identified longitudinal HbA1c patterns (Nguena Nguefack et al., 2020; Nagin, 2014). Two- to seven-group models were fitted using a censored normal distribution with quadratic time terms. Model selection considered BIC, classification quality (AvePP ≥0.70), group size, parsimony, and clinical interpretability rather than statistical fit alone. Quadratic terms captured non-linearity without subject-specific random effects or spline knots. Details are provided in Supplementary Table S1.

Baseline characteristics were summarized across trajectory groups as mean (SD), median (IQR), or n (%), as appropriate. Differences among trajectory groups were assessed using one-way analysis of variance or Kruskal–Wallis tests for continuous variables and chi-square tests for categorical variables.

In the primary analysis, multivariable multinomial logistic regression was used to examine baseline factors associated with trajectory membership, with the stable glycemic control group as the reference. Results are reported as relative risk ratios (RRRs) with 95% confidence intervals (CIs). Multivariable binary logistic regression was used to examine associations between trajectory membership and cumulative incident microvascular and macrovascular complications, with results reported as odds ratios (ORs) and 95% CIs. Models were adjusted for age, sex, BMI, SBP, DBP, eGFR, LDL-C, HDL—C, triglycerides, smoking status, alcohol use, comorbidity burden, healthcare coverage, number of antidiabetic drug groups, and baseline insulin use.

Missing baseline covariate data were addressed using multiple imputation by chained equations under the missing-at-random assumption. Twenty imputed datasets were generated, and estimates were combined using Rubin's rules (Sterne et al., 2009). The extent of missingness and variable-specific imputation methods are presented in Supplementary Table S2.

### 2.7 Sensitivity analyses

Complete-case analyses assessed the robustness of the primary multiple-imputation results. To minimize temporal overlap and potential reverse causation, a three-year landmark analysis was conducted. HbA1c trajectories were independently re-estimated using ≥3 valid measurements within three years after the index date, applying the same GBTM framework and selection criteria as the primary analysis.

The landmark was set at three years after the index date. Patients with the corresponding complication recorded on or before the landmark were excluded from the outcome-specific risk set. Only the first post-landmark complication was counted as an event; otherwise, participants were censored at the end of available follow-up, no later than December 31, 2025.

Multivariable Cox regression assessed associations between three-year trajectories and subsequent microvascular and macrovascular complications. Where proportional hazards assumptions were violated, extended Cox models with trajectory-by-log (time since landmark) interactions allowed time-varying associations. Adjusted hazard ratios (aHRs) and 95% CIs were estimated at one and three years post-landmark, adjusting for the same baseline covariates as the primary analysis. The same multiple-imputation approach was applied, with complete-case analyses assessing robustness.

All analyses were conducted using Stata/MP version 17.0 (StataCorp LLC, College Station, TX, USA) with the traj plugin. Two-sided *P* < 0.05 was considered statistically significant.

The study protocol was approved by the Institutional Review Board of Ubon Ratchathani University (Approval No. UBU-REC-21/2569). The requirement for informed consent was waived because the study used de-identified secondary administrative data.

## 3 Results

### 3.1 Cohort characteristics and HbA1c trajectories

Among 575,756 adults with incident T2D identified during 2017–2019, 142,687 were included in the analytic cohort (Fig. 1). Exclusions were due to no pharmacological treatment (*n* = 240,127), < 4 valid HbA1c measurements or < 5 years of follow-up (*n* = 164,483), and no recorded HbA1c measurement (*n* = 28,459). Among 547,297 individuals with ≥ 1 valid HbA1c measurement, 80.02% had ≥ 4 measurements before applying the five-year follow-up criterion (Supplementary Table S2, Panel B)**.**

**Fig. 1:**
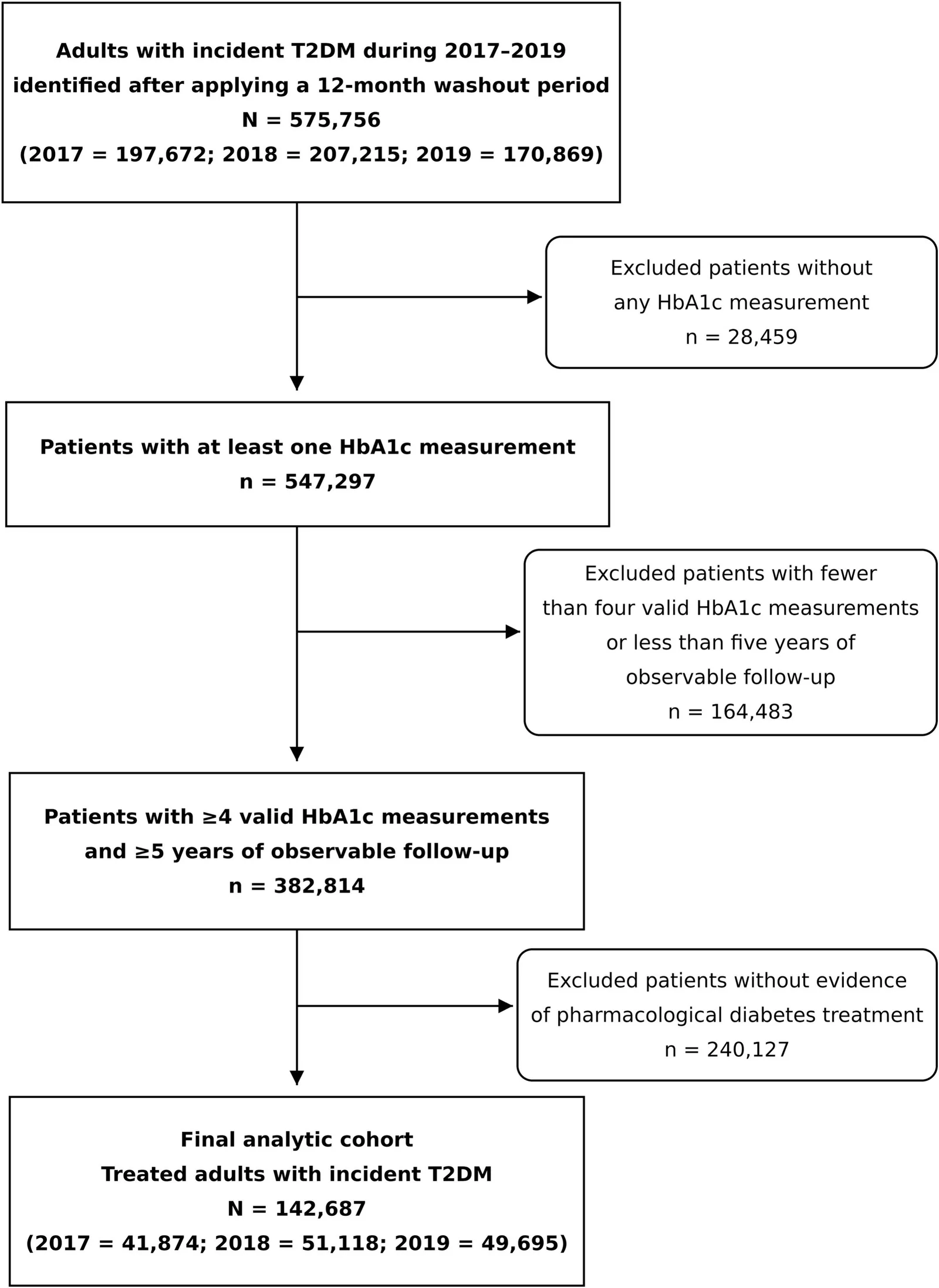
Flow diagram of cohort selection among adults with incident type 2 diabetes diagnosed in 2017–2019 and followed through 2025 in Thailand.

GBTM identified four distinct 5-year HbA1c trajectories (Fig. 2): stable glycemic control (Group 1; 59.39%), poor control with partial improvement (Group 2; 28.36%), very poor control with deterioration (Group 3; 6.62%), and persistently extremely poor control (Group 4; 5.63%). The four-group model provided the optimal balance of statistical fit, classification quality, parsimony, and clinical interpretability (Supplementary Table S1).

**Fig. 2:**
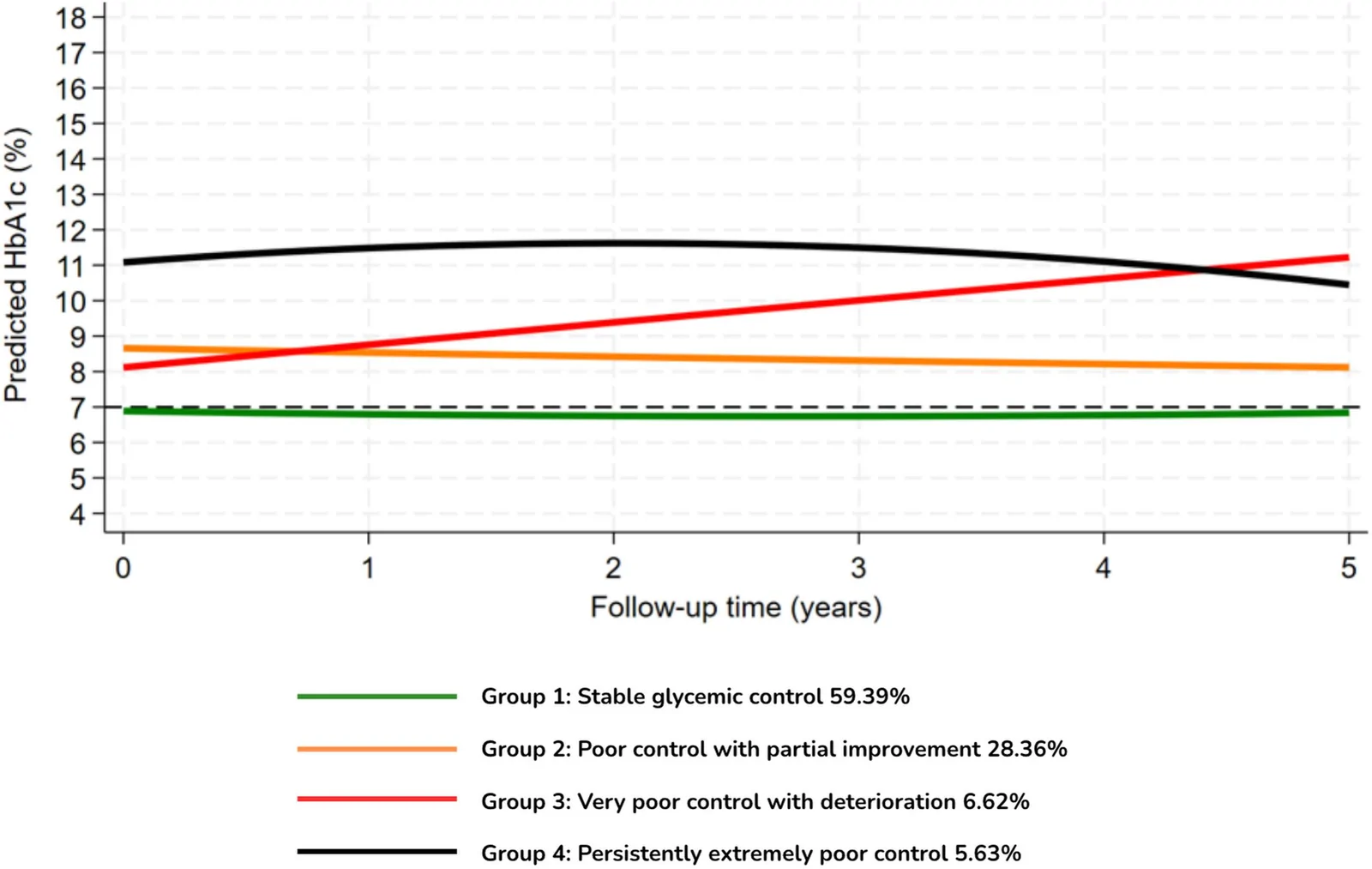
Five-year predicted HbA1c trajectories among adults with incident type 2 diabetes diagnosed in 2017–2019 and followed through 2025 in Thailand. Group 1, stable glycemic control (59.39%); Group 2, poor control with partial improvement (28.36%); Group 3, very poor control with deterioration (6.62%); and Group 4, persistently extremely poor control (5.63%). The dashed horizontal line indicates an HbA1c level of 7.0%. Model-estimated HbA1c values and corresponding 95% confidence intervals at each year of follow-up are presented in Supplementary Table S1 Panel C.

Baseline characteristics differed across trajectory groups (Table 1). Participants in unfavorable trajectory groups were generally younger and had higher baseline HbA1c, LDL-C, triglycerides, and eGFR. Baseline insulin use increased progressively across trajectory groups, from 17.42% in Group 1 to 69.64% in Group 4.

**Table 1:** Baseline characteristics by HbA1c trajectory group among adults with incident type 2 diabetes diagnosed in 2017–2019 and followed through 2025 in Thailand.

Characteristics	Overall (*N* = 142,687)	Group 1 (*n* = 84,737)	Group 2 (*n* = 40,472)	Group 3 (*n* = 9451)	Group 4 (*n* = 8027)	*P* value
		Stable glycemic control	Poor control with partial improvement	Very poor control with deterioration	Persistently extremely poor control	
Number of patients, n (%)	142,687 (100.00)	84,737 (59.39)	40,472 (28.36)	9451 (6.62)	8027 (5.63)	–
Age, years^a^, mean (SD)	54.64 (10.75)	56.78 (10.45)	52.56 (10.14)	49.84 (10.47)	48.11 (10.60)	< 0.01
Sex^c^, n (%)						< 0.01
- Male	60,077 (42.10)	36,709 (43.32)	16,223 (40.08)	3943 (41.72)	3202 (39.89)	
- Female	82,610 (57.90)	48,028 (56.68)	24,249 (59.92)	5508 (58.28)	4825 (60.11)	
Smoking status^c^, n (%)						< 0.01
- Non-smoker	124,550 (95.88)	73,538 (96.04)	35,661 (95.73)	8269 (95.47)	7082 (95.53)	
- Smoker	5349 (4.12)	3035 (3.96)	1591 (4.27)	392 (4.53)	331 (4.47)	
Alcohol use^c^, n (%)						0.01
- No	121,960 (94.77)	71,607 (94.62)	35,125 (94.91)	8208 (95.02)	7020 (95.35)	
- Yes	6728 (5.23)	4074 (5.38)	1882 (5.09)	430 (4.98)	342 (4.65)	
BMI, kg/m^2a^, mean (SD)	25.94 (4.73)	25.78 (4.62)	26.28 (4.79)	26.26 (5.02)	25.43 (5.12)	< 0.01
SBP, mmHg^a^, mean (SD)	120.94 (15.44)	121.59 (15.83)	120.32 (14.99)	119.43 (14.67)	118.90 (13.93)	< 0.01
DBP, mmHg^a^, mean (SD)	74.91 (9.46)	74.94 (9.49)	74.94 (9.45)	74.89 (9.42)	74.42 (9.22)	< 0.01
Baseline HbA1c^a^, % mean (SD)	7.96 (2.12)	7.08 (1.47)	9.07 (2.10)	8.30 (1.83)	11.30 (2.27)	< 0.01
eGFR, mL/min/1.73 m^2b^, mean (SD)	91.21 (23.17)	87.77 (22.72)	94.86 (22.64)	97.35 (23.26)	101.92 (22.93)	< 0.01
LDL-C, mg/dL^a^, mean (SD)	116.01 (40.96)	112.54 (39.97)	120.40 (41.65)	118.86 (41.15)	127.13 (43.13)	< 0.01
HDL-C, mg/dL^a^, mean (SD)	48.31 (12.83)	48.44 (12.80)	47.96 (12.63)	47.88 (13.01)	49.19 (13.85)	< 0.01
Triglycerides, mg/dL^b^, median (IQR)	148.00 (106.00–211.00)	143.00 (104.00–202.00)	156.00 (112.00–223.00)	155.00 (110.00–223.00)	156.00 (107.00–229.00)	< 0.01
Number of antidiabetic drug groups at baseline, n (%)						< 0.01
- 1 drug group	104,080 (72.94)	65,611 (77.43)	27,721 (68.49)	6084 (64.37)	4664 (58.10)	
- 2 drug groups	33,846 (23.72)	17,092 (20.17)	11,098 (27.42)	2907 (30.76)	2749 (34.25)	
- ≥ 3 drug groups	4761 (3.34)	2034 (2.40)	1653 (4.08)	460 (4.87)	614 (7.65)	
Insulin use at baseline^c^, n (%)						< 0.01
- No	103,501 (72.54)	69,976 (82.58)	26,341 (65.08)	4747 (50.23)	2437 (30.36)	
- Yes	39,186 (27.46)	14,761 (17.42)	14,131 (34.92)	4704 (49.77)	5590 (69.64)	
Comorbidity burden at baseline^c^, n (%)						< 0.01
- None	84,623 (59.31)	50,622 (59.74)	23,194 (57.31)	5606 (59.32)	5201 (64.79)	
- 1 comorbidity	35,046 (24.56)	19,935 (23.53)	10,788 (26.66)	2458 (26.01)	1865 (23.23)	
- ≥2 comorbidities	23,018 (16.13)	14,180 (16.73)	6490 (16.04)	1387 (14.68)	961 (11.97)	
Healthcare coverage scheme^c^, n (%)						< 0.01
Universal Coverage Scheme (UCS)	54,076 (37.90)	27,813 (32.82)	17,453 (43.12)	4647 (49.17)	4163 (51.86)	
Social Security Scheme (SSS)	15,561 (10.91)	8508 (10.04)	4776 (11.80)	1211 (12.81)	1066 (13.28)	
Civil Servant Medical Benefit Scheme (CSMBS)	19,670 (13.79)	13,567 (16.01)	4694 (11.60)	760 (8.04)	649 (8.09)	
Welfare/private insurance	52,207 (36.59)	34,156 (40.31)	13,201 (32.62)	2761 (29.21)	2089 (26.02)	
Other	1173 (0.82)	693 (0.82)	348 (0.86)	72 (0.76)	60 (0.75)	

**Abbreviations:** BMI, body mass index; DBP, diastolic blood pressure; eGFR, estimated glomerular filtration rate; HbA1c, glycated hemoglobin; HDL—C, high-density lipoprotein cholesterol; IQR, interquartile range; LDL-C, low-density lipoprotein cholesterol; SBP, systolic blood pressure; SD, standard deviation.

**Note:** Values are presented as mean (SD), median (IQR), or n (%), as appropriate. Baseline characteristics were summarized using the observed value closest to the index date for each variable and patient. Denominators may vary across variables because of missing data. Missing covariate information is summarized in Supplementary Table S2 (Panel A). Multiple imputation was applied to multivariable regression analyses but not to the descriptive baseline table. Comorbidity burden was categorized as 0, 1, or ≥ 2 baseline comorbid conditions identified from routinely collected clinical and diagnostic records before or within 1 year after the index date.

a One-way analysis of variance.

b Kruskal–Wallis test.

c Chi-square test.

### 3.2 Factors associated with HbA1c trajectory membership

In multivariable multinomial logistic regression (Table 2), younger age, female sex, smoking history, higher LDL-C, higher triglycerides, higher eGFR, and greater antidiabetic treatment intensity were associated with unfavorable trajectory membership.

**Table 2:** Baseline factors associated with HbA1c trajectory group membership among adults with incident type 2 diabetes diagnosed in 2017–2019 and followed through 2025 in Thailand.Reference category: Group 1 (stable glycemic control).

Variable	Group 2 vs Group 1 RRR (95% CI)	Group 3 vs Group 1 RRR (95% CI)	Group 4 vs Group 1 RRR (95% CI)
Age	0.98 (0.97, 0.98)	0.95 (0.95, 0.96)	0.95 (0.94, 0.95)
Female sex	1.16 (1.13, 1.19)	1.10 (1.05, 1.15)	1.18 (1.12, 1.24)
BMI	1.01 (1.00, 1.01) a	1.00 (0.99, 1.01)	0.97 (0.96, 0.97)
SBP	1.00 (1.00, 1.00) a	1.00 (1.00, 1.00) a	1.00 (0.99, 1.00) a
DBP	1.00 (1.00, 1.01) a	1.01 (1.00, 1.01) a	1.00 (1.00, 1.00)
eGFR	1.01 (1.01, 1.01)	1.01 (1.01, 1.01)	1.02 (1.01, 1.02)
LDL-C	1.00 (1.00, 1.00) a	1.00 (1.00, 1.00) a	1.01 (1.01, 1.01)
HDL-C	1.00 (1.00, 1.00) a	1.00 (1.00, 1.00)	1.00 (1.00, 1.01) a
Triglycerides	1.00 (1.00, 1.00) a	1.00 (1.00, 1.00) a	1.00 (1.00, 1.00) a
Current/former smoking	1.15 (1.07, 1.24)	1.19 (1.05, 1.34)	1.21 (1.05, 1.39)
Alcohol use	0.88 (0.82, 0.94)	0.80 (0.71, 0.90)	0.75 (0.65, 0.85)
Comorbidity burden: 1 comorbidity	1.07 (1.04, 1.10)	0.99 (0.94, 1.04)	0.77 (0.72, 0.81)
Comorbidity burden: ≥2 comorbidities	0.94 (0.90, 0.97)	0.82 (0.77, 0.88)	0.61 (0.57, 0.66)
Social Security Scheme (SSS)	0.85 (0.81, 0.88)	0.76 (0.71, 0.81)	0.73 (0.68, 0.79)
Civil Servant Medical Benefit Scheme (CSMBS)	0.81 (0.77, 0.84)	0.59 (0.54, 0.64)	0.65 (0.59, 0.71)
Welfare/private insurance	0.98 (0.95, 1.01)	1.01 (0.95, 1.07)	1.00 (0.94, 1.07)
Other coverage	0.90 (0.78, 1.03)	0.72 (0.56, 0.93)	0.72 (0.54, 0.96)
Number of antidiabetic drug groups	1.28 (1.25, 1.31)	1.32 (1.27, 1.37)	1.47 (1.41, 1.53)
Baseline insulin use	2.34 (2.27, 2.41)	4.05 (3.86, 4.24)	8.89 (8.42, 9.38)

**Abbreviations:** BMI, body mass index; CI, confidence interval; DBP, diastolic blood pressure; eGFR, estimated glomerular filtration rate; HDL—C, high-density lipoprotein cholesterol; LDL-C, low-density lipoprotein cholesterol; RRR, relative risk ratio; SBP, systolic blood pressure.

**Note:** Relative risk ratios (RRRs) and 95% confidence intervals (CIs) were estimated using multivariable multinomial logistic regression with multiple imputation by chained equations (20 imputations) for missing covariate data. Group 1 (stable glycemic control) was the reference trajectory group. Reference categories for categorical variables were male sex, non-smoker, no alcohol use, no comorbidity, and Universal Coverage Scheme (UCS), as applicable. Models were adjusted for age, sex, smoking status, alcohol use, body mass index, systolic and diastolic blood pressure, estimated glomerular filtration rate, LDL-C, HDL—C, triglycerides, comorbidity burden, healthcare coverage scheme, number of antidiabetic drug groups, and baseline insulin use.

a Rounding to two decimal places may obscure whether the 95% confidence interval includes 1.00. For marked estimates, the unrounded confidence interval excludes 1.00.

Baseline insulin use showed the strongest association with unfavorable trajectories. Compared with Group 1, baseline insulin use was associated with RRRs of 2.34 (95% CI, 2.27, 2.41) for Group 2, 4.05 (95% CI, 3.86, 4.24) for Group 3, and 8.89 (95% CI, 8.42, 9.38) for Group 4. Having ≥2 comorbidities and coverage under the Social Security Scheme or Civil Servant Medical Benefit Scheme were associated with lower likelihoods of membership in unfavorable trajectory groups compared with the respective reference categories.

### 3.3 HbA1c trajectories and diabetes-related complications

Adjusted associations between five-year HbA1c trajectory membership and incident complications are presented in Table 3 (Panel A). Compared with Group 1, the odds of macrovascular complications increased progressively across unfavorable trajectory groups: Group 2, adjusted OR (aOR) 1.23 (95% CI, 1.17, 1.29); Group 3, aOR 1.29 (95% CI, 1.19, 1.40); and Group 4, aOR 1.56 (95% CI, 1.44, 1.70).

**Table 3:** Associations between HbA1c trajectories and diabetes-related complications among adults with incident type 2 diabetes diagnosed in 2017–2019 and followed through 2025 in Thailand: Primary and three-year landmark analyses.

Panel A. Primary analysis: Five-year HbA1c trajectories (Multivariable logistic regression)				
HbA1c trajectory group	Microvascular complications; aOR (95% CI)		Macrovascular complications; aOR (95% CI)	
Group 1: Stable glycemic control	Reference		Reference	
Group 2:				
Poor control with partial improvement	1.10 (1.06, 1.14)		1.23 (1.17, 1.29)	
Group 3:				
Very poor control with deterioration	0.99 (0.93, 1.06)		1.29 (1.19, 1.40)	
Group 4:				
Persistently extremely poor control	1.09 (1.01, 1.17)		1.56 (1.44, 1.70)	

**Panel B. Three-year landmark sensitivity analysis: Extended Cox regression**				

**HbA1c trajectory group**	**Microvascular complications**		**Macrovascular complications**	

	aHR at 1 year (95% CI)	aHR at 3 years (95% CI)	aHR at 1 year (95% CI)	aHR at 3 years (95% CI)
Group 1: Stable glycemic control	Reference	Reference	Reference	Reference
Group 2:				
Poor control with partial improvement	1.19 (1.14, 1.24)	1.16 (1.11, 1.22)	1.28 (1.21, 1.36)	1.28 (1.20, 1.35)
Group 3:				
Very poor control with deterioration	1.10 (1.01, 1.19)	1.09 (0.99, 1.20)	1.35 (1.21, 1.52)	1.58 (1.43, 1.74)
Group 4:				
Persistently extremely poor control	1.51 (1.40, 1.64)	1.52 (1.38, 1.65)	1.74 (1.57, 1.93)	2.18 (1.99, 2.37)

Abbreviations**:** HbA1c, glycated hemoglobin; aOR, adjusted odds ratio; aHR, adjusted hazard ratio; CI, confidence interval.

Notes**:** Panel A presents adjusted odds ratios from multivariable logistic regression models examining associations between five-year HbA1c trajectories and incident complications in the primary analytic cohort (n = 142,687).

Panel B presents time-specific adjusted hazard ratios from extended Cox regression models based on independently estimated three-year HbA1c trajectories (*n* = 120,270). The microvascular analysis included 87,824 participants and 13,513 post-landmark events; the macrovascular analysis included 106,748 participants and 8571 post-landmark events. Participants with the corresponding complication recorded on or before the three-year landmark were excluded from each outcome-specific risk set. Hazard ratios were estimated at one and three years after the landmark.

All models were adjusted for age, sex, body mass index, systolic and diastolic blood pressure, estimated glomerular filtration rate, LDL-C, HDL—C, triglycerides, smoking status, alcohol use, comorbidity burden, healthcare coverage, number of antidiabetic drug groups, and baseline insulin use. Missing baseline covariate data were addressed using multiple imputation with 20 imputed datasets.

Trajectory groups were independently estimated for the five-year and three-year analyses. Odds ratios and hazard ratios represent different measures of association and should not be compared directly.

For microvascular complications, Group 2 was associated with higher odds (aOR 1.10; 95% CI, 1.06, 1.14), as was Group 4 (aOR 1.09; 95% CI, 1.01, 1.17). No significant association was observed for Group 3 (aOR 0.99; 95% CI, 0.93, 1.06).

### 3.4 Sensitivity analyses

Complete-case analysis of the primary five-year trajectories (*n* = 105,173) yielded findings consistent with multiple imputation. Trajectory distributions were comparable between the full and complete-case cohorts (Supplementary Table S3). Baseline insulin use remained strongly associated with Group 4 membership (RRR 9.03; 95% CI, 8.48, 9.61; Supplementary Table S3). Macrovascular complication aORs were 1.25 (95% CI, 1.18, 1.31), 1.29 (1.18, 1.41), and 1.58 (1.44, 1.73) for Groups 2–4, respectively (Supplementary Table S4).

To address temporal overlap, three-year landmark analysis independently identified four trajectories among 120,270 participants with ≥3 valid HbA1c measurements: stable glycemic control (Group 1; *n* = 79,230, 65.88%), poor control with partial improvement (Group 2; *n* = 29,310, 24.37%), very poor control with deterioration (Group 3; *n* = 5754, 4.78%), and persistently extremely poor control (Group 4; *n* = 5976, 4.97%). Classification was satisfactory (entropy = 0.82; average posterior probabilities, 0.80–0.93; Supplementary Table S5 and Supplementary Fig. S1). After excluding participants with complications recorded on or before the three-year landmark, the outcome-specific risk sets comprised 87,824 participants for microvascular complications and 106,748 for macrovascular complications (Fig. 3).

**Fig. 3:**
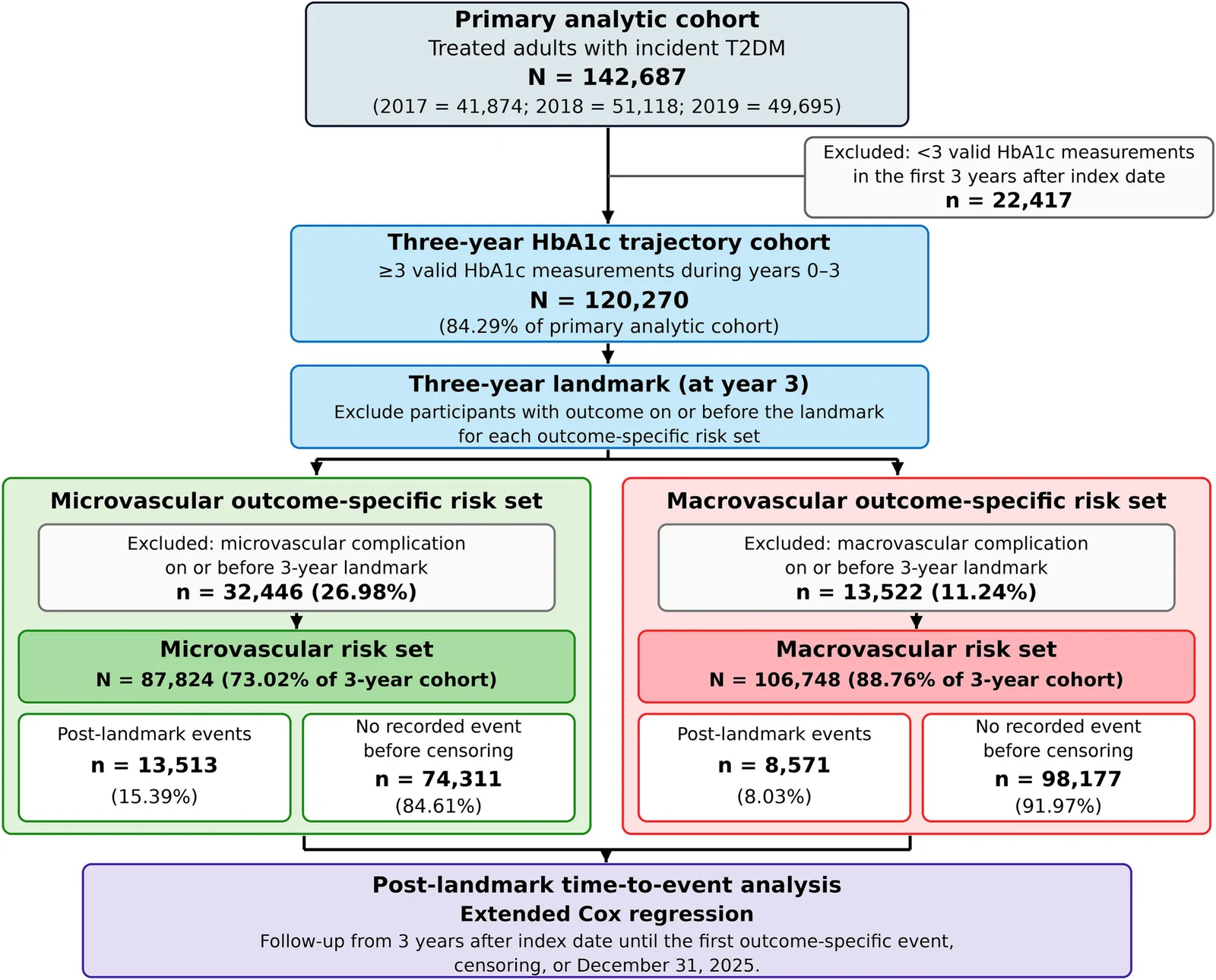
Flow diagram of cohort selection and outcome-specific risk sets for the three-year landmark analysis among adults with incident type 2 diabetes diagnosed in 2017–2019 and followed through 2025 in Thailand. Of the 142,687 participants in the primary analytic cohort, 120,270 (84.29%) had ≥3 valid HbA1c measurements during the first three years after diagnosis. After excluding participants with outcome-specific complications recorded on or before the three-year landmark, 87,824 (73.02%) and 106,748 (88.76%) participants were included in the microvascular and macrovascular risk sets, respectively. During post-landmark follow-up, 13,513 microvascular and 8571 macrovascular events were recorded. Associations were evaluated using extended Cox regression, with follow-up continuing until the first outcome-specific event, censoring, or December 31, 2025.

The microvascular landmark analysis included 87,824 participants (13,513 events). Compared with Group 1, aHRs for Groups 2–4 were 1.19 (95% CI, 1.14, 1.24), 1.10 (1.01, 1.19), and 1.51 (1.40, 1.64) at one year, and 1.16 (1.11, 1.22), 1.09 (0.99, 1.20), and 1.52 (1.38, 1.65) at three years, respectively. Trajectory-by-time interactions were nonsignificant (all *P* > 0.05; Table 3, Panel B).

The macrovascular analysis included 106,748 participants (8571 events). Corresponding aHRs were 1.28 (95% CI, 1.21, 1.36), 1.35 (95% CI, 1.21, 1.52), and 1.74 (95% CI, 1.57, 1.93) at one year, increasing to 1.28 (95% CI, 1.20, 1.35), 1.58 (95% CI, 1.43, 1.74), and 2.18 (95% CI, 1.99, 2.37) at three years. Trajectory-by-time interactions were significant for Groups 3 (*P* < 0.01) and 4 (*P* < 0.01), but not Group 2 (*P* = 0.85; Table 3, Panel B).

Complete-case landmark analyses yielded comparable estimates, including 65,686 participants (10,126 events) for microvascular and 79,801 (6409 events) for macrovascular outcomes. For Group 4, microvascular aHRs were 1.52 (95% CI, 1.39, 1.65) and 1.54 (1.39, 1.70) at one and three years, respectively; corresponding macrovascular aHRs were 1.64 (1.45, 1.85) and 2.12 (1.90, 2.33) (Supplementary Table S4).

## 4 Discussion

In this nationwide cohort of 142,687 Thai adults with incident T2D, four distinct 5-year HbA1c trajectories were identified. Nearly 60% maintained stable glycemic control, whereas over 40% followed unfavorable trajectories involving persistent hyperglycemia, partial improvement, or deterioration. Younger age and baseline insulin use were particularly associated with unfavorable trajectories. These trajectories showed graded associations with macrovascular complications, while microvascular associations were weaker and less consistent. Overall, these findings highlight substantial heterogeneity in long-term glycemic control and its association with clinical outcomes.

The four identified trajectories align with previous evidence of heterogeneous long-term glycemic control in T2D (Luo et al., 2018; Venkataraman et al., 2017; Walraven et al., 2015). Htun et al. also identified four distinct HbA1c trajectories despite differences in populations, settings, and follow-up periods (Htun et al., 2023). While previous Thai studies primarily examined cross-sectional glycemic control or characteristics at diagnosis (Puangpet et al., 2022; Techasuwanna et al., 2020; Preechasuk et al., 2022), our findings demonstrate the potential of nationwide routine data to characterize longitudinal glycemic patterns. Identifying groups with persistent glycemic vulnerability may support closer monitoring and earlier preventive interventions.

Younger age was associated with unfavorable glycemic trajectories, consistent with evidence of more aggressive younger-onset T2D (Lascar et al., 2018). Baseline insulin use showed the strongest association, particularly with persistently extremely poor control, potentially reflecting greater initial disease severity or marked hyperglycemia rather than an adverse insulin effect (American Diabetes Association, 2024). Higher LDL-C and triglycerides were also associated with unfavorable trajectories. The inverse association between BMI and the most unfavorable trajectory may reflect a greater contribution of β-cell dysfunction than obesity in some East Asian populations (Yabe et al., 2015; Kodama et al., 2013). These findings highlight broader cardiometabolic vulnerability and support integrated glycemic and cardiovascular prevention.

Female predominance in our cohort aligns with Thai data showing higher diabetes prevalence among women than men (10.80% vs. 8.90% in 2014) (Aekplakorn et al., 2018) and a nationwide cohort of 186,010 patients with T2D, of whom 68.50% were women. Female sex has also been associated with poor glycemic control in Thailand (Sakboonyarat et al., 2021).

The strongest outcome finding was a graded association between unfavorable HbA1c trajectories and macrovascular complications, with adjusted odds increasing across Groups 2–4 compared with stable glycemic control. This aligns with established evidence linking glycemic exposure to vascular complications (Farmaki et al., 2020; Stratton et al., 2000). In contrast, microvascular associations were weaker and non-monotonic, with no significant association for Group 3. Although these differences warrant cautious interpretation, the macrovascular gradient suggests that longitudinal glycemic patterns may help identify populations requiring sustained cardiovascular prevention and follow-up.

The three-year landmark analysis supported the primary findings by separating HbA1c trajectory assessment from subsequent complications. Associations persisted for both microvascular and macrovascular outcomes, particularly in Group 4. Macrovascular relative hazards increased over time in Groups 3 and 4, highlighting the potential relevance of unfavorable early glycemic trajectories to subsequent vascular risk.

The stronger associations with macrovascular than microvascular complications warrant caution. UKPDS 35 found a stronger association of glycemic exposure with microvascular outcomes than myocardial infarction (Stratton et al., 2000). Macrovascular disease is multifactorial, involving hypertension, dyslipidemia, and other cardiovascular risk factors (American Diabetes Association Professional Practice Committee for Diabetes, 2026). Some post-diagnosis events may reflect pre-existing subclinical disease despite excluding complications recorded before or within one year after diagnosis, given incomplete historical records. Reliance on ICD-10 diagnoses rather than systematic screening may also have underestimated microvascular complications. Disease latency, outcome ascertainment, and residual confounding may explain these findings without implying a stronger causal effect of glycemia on macrovascular disease.

The study has several strengths, including its nationwide population-based data, large sample size, incident-disease design, 5-year trajectory assessment, and sensitivity analyses using complete cases. However, several limitations should be considered.

First, requiring ≥5 years of follow-up may have introduced survivor selection by favoring individuals who survived and remained observable. Requiring ≥4 HbA1c measurements may have introduced informative selection, as testing frequency may reflect glycemic control, comorbidity, and healthcare utilization. Restricting the cohort to pharmacologically treated individuals further excluded those managed solely through lifestyle modification. Together, these criteria may limit generalizability to all individuals with newly diagnosed T2D.

Second, despite broad coverage, the HDC may underrepresent individuals receiving care outside the public healthcare system or with incomplete records. Although diagnosis and glucose-lowering medication records were used, the 12-month washout may not have excluded prevalent diabetes, and administrative diagnoses may have misclassified diabetes type. Reliance on ICD-10 diagnoses rather than systematic screening may have underestimated microvascular complications, while unavailable pre-2016 records may have caused pre-existing complications to be misclassified as incident events.

Third, the primary analysis used cumulative binary outcomes without accounting for event timing or time-varying treatments (Hosmer et al., 2013; Rothman et al., 2021). Overlapping five-year trajectory and complication assessments allowed potential reverse causation. Although the three-year landmark analysis separated trajectory assessment from subsequent events using time-to-event models, pre-existing subclinical disease could still cause reverse causation. Competing-risk analysis for death was infeasible without reliable mortality data.

Longitudinal HbA1c data from routine care can reveal clinically and epidemiologically meaningful glycemic heterogeneity beyond single measurements. Identifying persistent or worsening trajectories may complement monitoring and inform earlier population-level prevention, particularly cardiovascular risk reduction. Prospective studies should clearly separate trajectory assessment from outcomes before applying these approaches to individual-level clinical prediction.

## 5 Conclusions

In this nationwide cohort of adults with incident T2D in Thailand, unfavorable 5-year HbA1c trajectories were common and associated with higher odds of diabetes-related complications, particularly macrovascular disease. Routinely collected longitudinal HbA1c data may identify populations with sustained glycemic vulnerability and inform earlier prevention and cardiovascular risk reduction. Prospective studies clearly separating trajectory assessment from subsequent outcomes are needed before applying trajectory-based approaches to individual-level clinical prediction.

## Generative AI usage

During the preparation of this work, the authors used ChatGPT (OpenAI) to assist with the language flow and readability of the manuscript. After using this tool, the authors reviewed and edited the content as needed and take full responsibility for the content of the published article.

## Studies in human

This study was performed in compliance with relevant laws, regulatory frameworks and guidelines where the research took place.

This study was approved by the Institutional Review Board of Ubon Ratchathani University.

(Approval No. UBU-REC-21/2569)

## CRediT authorship contribution statement

**Anuchit Sermhattakit:** Writing – review & editing, Writing – original draft, Methodology, Formal analysis, Data curation, Conceptualization. **Sawaeng Watcharathanakij:** Writing – review & editing, Supervision, Methodology, Data curation, Conceptualization.

## Ethics declaration

### Informed consent and patient details

The authors have NOT obtained informed consent from participants or their legal representatives. The requirement for informed consent was waived by the Institutional Review Board of Ubon Ratchathani University (Approval No. UBU-REC-21/2569) because this study was a retrospective cohort analysis using fully de-identified secondary administrative electronic medical records, posing minimal risk to study participants.

## Funding

This study was supported by a Doctoral Dissertation Grant from the Faculty of Pharmaceutical Sciences, Ubon Ratchathani University, Thailand. The funder had no role in the study design, data collection and analysis, decision to publish, or preparation of the manuscript.

## Declaration of competing interest

The authors declare that they have no known competing financial interests or personal relationships that could have appeared to influence the work reported in this paper.

## Data Availability

The authors do not have permission to share data.
