# Supplementary material for: Long-term HbA1c trajectories and diabetes-related complications among adults with incident type 2 diabetes: A nationwide cohort study in Thailand
Source: Prev Med Rep. 2026 Nov 5;71:103693. doi: 10.1016/j.pmedr.2026.103693 (PMC13770716; doi:10.1016/j.pmedr.2026.103693)
Supplement: Supplementary file 1 [file mmc1.docx]

**Supplementary Table S1.**

Model selection, classification performance, and estimated HbA1c trajectories among adults with incident type 2 diabetes diagnosed in 2017–2019 and followed through 2025 in Thailand.

| **Panel A**. Model Selection Criteria (2-7 Trajectory groups) | | | | | | | | | |
| --- | --- | --- | --- | --- | --- | --- | --- | --- | --- |
| Number  of groups | Polynomial order | BIC | AIC | | Entropy | Smallest group (%) | | Model Evaluation | |
| 2 | 22 | −1,294,390.06 | −1,294,350.59 | | 0.88 | 21.54 | | High classification certainty and excellent entropy; however, the model oversimplified glycemic heterogeneity and failed to distinguish clinically meaningful trajectory patterns. | |
| 3 | 222 | −1,268,268.18 | −1,268,208.97 | | 0.81 | 8.21 | | Improved trajectory differentiation; however, intermediate patterns remained insufficiently separated for clinical interpretation. | |
| 4 | 2222 | −1,258,507.32 | −1,258,428.37 | | 0.81 | 5.63 | | Selected model. Provided clinically interpretable trajectory patterns with satisfactory classification performance (entropy = 0.81), adequate subgroup sizes (smallest group = 5.63%), and greater parsimony than models with additional groups. | |
| 5 | 22222 | −1,247,419.50 | −1,247,320.82 | | 0.76 | 3.93 | | Additional trajectory groups provided limited clinical information and resulted in smaller subgroup sizes with reduced classification certainty. | |
| 6 | 222222 | −1,240,683.65 | −1,240,565.23 | | 0.78 | 3.26 | | Increasing model complexity yielded minimal improvement in clinical interpretability and introduced potential instability due to small subgroup membership. | |
| 7 | 2222222 | −1,236,291.65 | −1,236,153.49 | | 0.78 | 2.74 | | Most complex solution with potential overfitting, reduced classification quality, and very small trajectory groups that may limit generalizability. | |
| **Panel B**. Classification performance of the selected four-group model | | | | | | | | | |
| Trajectory group | | Group description | | | Group size, n (%) | | | Average posterior probability (AvePP) | |
| Group 1 | | Stable glycemic control | | | 84,737 (59.39) | | | 0.92 | |
| Group 2 | | Poor control  with partial improvement | | | 40,472 (28.36) | | | 0.83 | |
| Group 3 | | Very poor control with deterioration | | | 9,451 (6.62) | | | 0.83 | |
| Group 4 | | Persistently extremely poor control | | | 8,027 (5.63) | | | 0.90 | |
| **Panel C.** Model-estimated HbA1c trajectories and 95% confidence intervals | | | | | | | | | |
| Follow-up | | Group 1 | | Group 2 | | | Group 3 | | Group 4 |
| 0 year | | 6.89 (6.88, 6.90) | | 8.66 (8.64, 8.68) | | | 8.12 (8.08, 8.16) | | 11.08 (11.04, 11.12) |
| 1 year | | 6.80 (6.79, 6.81) | | 8.54 (8.52, 8.55) | | | 8.76 (8.72, 8.79) | | 11.48 (11.45, 11.51) |
| 2 years | | 6.75 (6.74, 6.76) | | 8.42 (8.40, 8.44) | | | 9.39 (9.35, 9.43) | | 11.62 (11.59, 11.65) |
| 3 years | | 6.74 (6.73, 6.75) | | 8.31 (8.30, 8.33) | | | 10.01 (9.97, 10.05) | | 11.49 (11.46, 11.52) |
| 4 years | | 6.77 (6.76, 6.78) | | 8.21 (8.20, 8.23) | | | 10.62 (10.58, 10.66) | | 11.10 (11.07, 11.13) |
| 5 years | | 6.84 (6.83, 6.85) | | 8.12 (8.10, 8.14) | | | 11.23 (11.17, 11.29) | | 10.45 (10.40, 10.50) |

**Abbreviations:** AIC, Akaike information criterion; BIC, Bayesian information criterion; AvePP, average posterior probability; CI, confidence interval.

Notes: Models with 2–7 trajectory groups were evaluated using a censored normal distribution with quadratic polynomial terms. Although BIC and AIC improved with increasing model complexity, the four-group model was selected based on classification quality, adequate subgroup sizes, parsimony, and clinical interpretability rather than statistical fit alone. An AvePP ≥ 0.70 indicated adequate classification. In Panel C, follow-up time represents years since the index date (0 = index date; 1–5 = years after the index date). Values are model-estimated HbA1c (%) at each specified time point, with 95% confidence intervals in parentheses.

**Supplementary Table S2.**

Baseline covariate missingness, imputation methods, and HbA1c measurement frequency among adults with incident type 2 diabetes diagnosed in 2017–2019 in Thailand, using data through 2025.

| **Panel A**. Baseline Covariate Missingness and Imputation Methods (N = 142,687) | | | | | | |
| --- | --- | --- | --- | --- | --- | --- |
| Covariate | Observed, n | Missing, n | | Missing, % | Imputation method | |
| Smoking status | 129,899 | 12,788 | | 8.96 | Logistic regression | |
| Alcohol use | 128,688 | 13,999 | | 9.81 | Logistic regression | |
| Baseline systolic blood pressure | 141,823 | 864 | | 0.61 | Linear regression | |
| Baseline diastolic blood pressure | 141,735 | 952 | | 0.67 | Linear regression | |
| Baseline eGFR | 139,191 | 3,496 | | 2.45 | Linear regression | |
| Baseline LDL cholesterol | 140,871 | 1,816 | | 1.27 | Linear regression | |
| Baseline HDL cholesterol | 130,927 | 11,760 | | 8.24 | Linear regression | |
| Baseline triglycerides | 136,480 | 6,207 | | 4.35 | Linear regression | |
| Baseline body mass index | 132,767 | 9,920 | | 6.95 | Linear regression | |
| **Panel B**. Distribution of Valid HbA1c Measurement Frequency (N = 547,297) | | | | | | |
| Valid HbA1c measurements | | | n | | | % |
| 1 | | | 41,786 | | | 7.63 |
| 2 | | | 34,937 | | | 6.38 |
| 3 | | | 32,636 | | | 5.96 |
| ≥ 4 | | | 437,938 | | | 80.02 |
| Total | | | 547,297 | | | 100.00 |

Notes:

Panel A: Missingness was assessed at the participant level in the analytic cohort of 142,687 participants. Baseline covariates were defined using measurements within ± 6 months of the index date, with the value closest to the index date selected when multiple measurements were available. Missing baseline covariate data were handled using multiple imputation by chained equations under the missing-at-random assumption, with 20 imputed datasets generated.

Panel B: Percentages were calculated among 547,297 individuals remaining after exclusion of those with no recorded HbA1c measurement, corresponding to the first “Remaining participants” step in Figure 1. The ≥4 category reflects HbA1c measurement frequency only and does not account for the ≥ 5-year follow-up criterion.

**Supplementary Table S3.**

Comparison of HbA1c trajectory group distributions and associated factors between multiple-imputation and complete-case analyses among adults with incident type 2 diabetes diagnosed in 2017–2019 and followed through 2025 in Thailand.

| **Panel A**. HbA1c Trajectory Group Distributions | | | |
| --- | --- | --- | --- |
| HbA1c trajectory group | | Full analytic cohort  (N=142,687), n (%) | Complete-case cohort  (N=105,173), n (%) |
| Group 1: Stable glycemic control | | 84,737 (59.39) | 61,148 (58.14) |
| Group 2: Poor control with partial improvement | | 40,472 (28.36) | 30,683 (29.17) |
| Group 3: Very poor control with deterioration | | 9,451 (6.62) | 7,391 (7.03) |
| Group 4: Persistently extremely poor control | | 8,027 (5.63) | 5,951 (5.66) |
| Total | | 142,687 (100.00) | 105,173 (100.00) |
| **Panel B**. Factors Associated with HbA1c Trajectory Group Membership | | | |
| Predictor | Comparison | Multiple imputation RRR  (95% CI) | Complete-case RRR  (95% CI) |
| Age (per year) | Group 2 vs 1 | 0.98 (0.97, 0.98) | 0.98 (0.97, 0.98) |
|  | Group 3 vs 1 | 0.95 (0.95, 0.96) | 0.95 (0.95, 0.96) |
|  | Group 4 vs 1 | 0.95 (0.94, 0.95) | 0.95 (0.94, 0.95) |
| Female sex | Group 2 vs 1 | 1.16 (1.13, 1.19) | 1.16 (1.13, 1.20) |
|  | Group 3 vs 1 | 1.10 (1.05, 1.15) | 1.12 (1.06, 1.18) |
|  | Group 4 vs 1 | 1.18 (1.12, 1.24) | 1.22 (1.14, 1.30) |
| Current/former smoking | Group 2 vs 1 | 1.15 (1.07, 1.24) | 1.16 (1.07, 1.25) |
|  | Group 3 vs 1 | 1.19 (1.05, 1.34) | 1.20 (1.04, 1.38) |
|  | Group 4 vs 1 | 1.21 (1.05, 1.39) | 1.15 (0.98, 1.34) |
| Alcohol use | Group 2 vs 1 | 0.88 (0.82, 0.94) | 0.89 (0.83, 0.96) |
|  | Group 3 vs 1 | 0.80 (0.71, 0.90) | 0.82 (0.72, 0.93) |
|  | Group 4 vs 1 | 0.75 (0.65, 0.85) | 0.75 (0.65, 0.87) |
| Number of antidiabetic drug groups | Group 2 vs 1 | 1.28 (1.25, 1.31) | 1.27 (1.23, 1.30) |
|  | Group 3 vs 1 | 1.32 (1.27, 1.37) | 1.32 (1.26, 1.38) |
|  | Group 4 vs 1 | 1.47 (1.41, 1.53) | 1.44 (1.38, 1.51) |
| Baseline insulin use | Group 2 vs 1 | 2.34 (2.27, 2.41) | 2.28 (2.21, 2.36) |
|  | Group 3 vs 1 | 4.05 (3.86, 4.24) | 4.12 (3.92, 4.35) |
|  | Group 4 vs 1 | 8.89 (8.42, 9.38) | 9.03 (8.48, 9.61) |

Abbreviations: CI, confidence interval; RRR, relative risk ratio.

Notes: Panel A compares assigned trajectory membership in the full analytic cohort and complete-case cohort. Panel B presents selected predictors from multivariable multinomial logistic regression, with Group 1 (stable glycemic control) as the reference. Multiple imputation used 20 imputed datasets (N=142,687); the complete-case analysis included 105,173 participants with complete baseline covariate data. Both models included the same covariates as specified in the primary analysis.

**Supplementary Table S4.**

Comparison of multiple-imputation and complete-case estimates for associations between HbA1c trajectories and diabetes-related complications among adults with incident type 2 diabetes diagnosed in 2017–2019 and followed through 2025 in Thailand.

**Panel A.** Primary analysis: Five-year HbA1c trajectories (multivariable logistic regression)

| Outcome | Analysis | N | Group 2  aOR (95% CI) | Group 3  aOR (95% CI) | Group 4  aOR (95% CI) |
| --- | --- | --- | --- | --- | --- |
| Microvascular complications | MI | 142,687 | 1.10 (1.06, 1.14) | 0.99 (0.93, 1.06) | 1.09 (1.01, 1.17) |
|  | Complete-case | 105,173 | 1.11 (1.07, 1.16) | 0.97 (0.90, 1.05) | 1.13 (1.04, 1.22) |
| Macrovascular complications | MI | 142,687 | 1.23 (1.17, 1.29) | 1.29 (1.19, 1.40) | 1.56 (1.44, 1.70) |
|  | Complete-case | 105,173 | 1.25 (1.18, 1.31) | 1.29 (1.18, 1.41) | 1.58 (1.44, 1.73) |

**Panel B**. Three-year landmark analysis: Post-landmark time-to-event outcomes (extended Cox regression)

| Outcome / Time point | Analysis | N (events) | Group 2  aHR (95% CI) | Group 3  aHR (95% CI) | Group 4  aHR (95% CI) |
| --- | --- | --- | --- | --- | --- |
| Microvascular complications |  |  |  |  |  |
| At 1 year | MI | 87,824 (13,513) | 1.19 (1.14, 1.24) | 1.10 (1.01, 1.19) | 1.51 (1.40, 1.64) |
|  | Complete-case | 65,686 (10,126) | 1.17 (1.11, 1.23) | 1.09 (0.99, 1.20) | 1.52 (1.39, 1.65) |
| At 3 years | MI | 87,824 (13,513) | 1.16 (1.11, 1.22) | 1.09 (0.99, 1.20) | 1.52 (1.38, 1.65) |
|  | Complete-case | 65,686 (10,126) | 1.15 (1.09, 1.22) | 1.07 (0.95, 1.19) | 1.54 (1.39, 1.70) |
| Macrovascular complications |  |  |  |  |  |
| At 1 year | MI | 106,748 (8,571) | 1.28 (1.21, 1.36) | 1.35 (1.21, 1.52) | 1.74 (1.57, 1.93) |
|  | Complete-case | 79,801 (6,409) | 1.26 (1.17, 1.35) | 1.31 (1.15, 1.49) | 1.64 (1.45, 1.85) |
| At 3 years | MI | 106,748 (8,571) | 1.28 (1.20, 1.35) | 1.58 (1.43, 1.74) | 2.18 (1.99, 2.37) |
|  | Complete-case | 79,801 (6,409) | 1.25 (1.17, 1.34) | 1.55 (1.38, 1.73) | 2.12 (1.90, 2.33) |

Abbreviations**:** HbA1c, glycated hemoglobin; MI, multiple imputation; aOR, adjusted odds ratio; aHR, adjusted hazard ratio; CI, confidence interval; N, number of participants.

Notes:

Group 1 (stable glycemic control) was the reference category in all analyses.

Panel A presents adjusted odds ratios and 95% confidence intervals from multivariable logistic regression models based on five-year HbA1c trajectories. The multiple-imputation analysis included 142,687 participants, whereas the complete-case analysis included 105,173 participants.

Panel B presents time-specific adjusted hazard ratios and 95% confidence intervals from extended Cox regression models based on independently estimated three-year HbA1c trajectories. A fixed landmark was established at three years after the index date, and participants with the corresponding complication recorded on or before the landmark were excluded from the outcome-specific risk set. Hazard ratios were estimated at one and three years after the landmark using trajectory-by-log(time since the landmark) interactions. The numbers of events represent total post-landmark events during the available follow-up, rather than events occurring exclusively at the specified time points.

All models were adjusted for age, sex, body mass index, systolic and diastolic blood pressure, estimated glomerular filtration rate, low-density and high-density lipoprotein cholesterol, triglycerides, smoking status, alcohol use, comorbidity burden, healthcare coverage, number of antidiabetic drug groups, and baseline insulin use. Multiple imputation was performed using 20 imputed datasets, and estimates were combined using Rubin’s rules.

Trajectory groups were independently estimated for the five-year primary analysis and the three-year landmark analysis. Odds ratios and hazard ratios represent different measures of association and should not be compared directly.

**Supplementary Table S5.**

Three-year HbA1c trajectory group characteristics and model classification performance in a landmark analysis among adults with incident type 2 diabetes diagnosed in 2017–2019 and followed through 2025 in Thailand.

| Trajectory group | n (%) | AvePP |
| --- | --- | --- |
| Group 1: Stable glycemic control | 79,230 (65.88) | 0.93 |
| Group 2: Poor control with partial improvement | 29,310 (24.37) | 0.81 |
| Group 3: Very poor control with deterioration | 5,754 (4.78) | 0.80 |
| Group 4: Persistently extremely poor control | 5,976 (4.97) | 0.90 |
| Total | 120,270 (100.00) | — |

Model classification entropy: 0.82

Model parameter estimates

| Trajectory group | Intercept | Linear | Quadratic |
| --- | --- | --- | --- |
| Group 1 | 6.94 | −0.19 | 0.06 |
| Group 2 | 8.95 | −0.38 | 0.05 |
| Group 3 | 8.14 | 0.49 | 0.21 |
| Group 4 | 11.23 | 0.69 | −0.26 |

Model fit: BIC = −777,627.28; AIC = −777,549.70; entropy = 0.82.

Abbreviations: HbA1c, glycated hemoglobin; AvePP, average posterior probability; BIC, Bayesian information criterion; AIC, Akaike information criterion.

Note: HbA1c trajectories were independently re-estimated using measurements obtained during the first three years after T2DM diagnosis among participants with at least three valid HbA1c measurements (N = 120,270). Group sizes and percentages were based on assigned trajectory membership. Average posterior probabilities (AvePPs) ranged from 0.80 to 0.93, and overall classification entropy was 0.82, indicating satisfactory classification performance. The trajectory model included linear and quadratic time terms.

**Supplementary Figure S1.**

Three-year model-estimated HbA1c trajectories among adults with incident type 2 diabetes diagnosed in 2017–2019 in Thailand.

Model-estimated HbA1c trajectories during the first three years after T2DM diagnosis among 120,270 participants with at least three valid HbA1c measurements. Four distinct trajectory groups were identified: stable glycemic control (65.88%), poor control with partial improvement (24.37%), very poor control with deterioration (4.78%), and persistently extremely poor control (4.97%). The dashed horizontal line indicates an HbA1c level of 7.0%.
